# The Effects of Parenting and Temperament Similarity Among Adolescent Siblings on Positive Family Relationships

**DOI:** 10.3389/fpsyg.2021.702000

**Published:** 2021-07-28

**Authors:** Xinxin Shi, Nicole Campione-Barr

**Affiliations:** ^1^School of Education, Zhengzhou University, Zhengzhou, China; ^2^School of Marxism, Zhengzhou University, Zhengzhou, China; ^3^Department of Psychological Sciences, University of Missouri, Columbia, MO, United States

**Keywords:** parenting, temperament, sibling relationships, family relationship, parent-adolescent relationships

## Abstract

The detrimental effects of parental differential treatment have been shown in previous research, but fewer researchers have pointed out that differential treatment does not always lead to negative outcomes. Thus, the present study examines the role of temperament similarity on the association between parenting similarity and positive family relationship qualities over 1 year in 145 adolescent sibling dyads (*M*_first−born_ = 14.97 and *SD* = 1.68 years; *M*_second−born_ = 12.20 and *SD* = 1.92 years). Latent moderator structure models (LMS) showed that a higher level of parenting similarity was related to more positive family relationships when siblings were more similar in their temperaments; however, a lower level of parenting similarity was related to more positive relationship qualities with family members 1 year later in the context of less sibling temperament similarity.

## Introduction

Adolescence is a special transitional period from childhood to adulthood. The rapid biological and cognitive development can lead to temporary perturbations in interpersonal relationships, which can also lead to emotional problems (Schwartz et al., 2015), and the potential development of the health of adolescents (Pompili et al., 2012). Empirical studies point out that support from family members is of real importance for adolescents as those with better relationships with parents and siblings are less likely to suffer from these emotional and behavioral problems (Buist, 2010; Shelton and Bree, 2010; Caputi et al., 2017). From a family systems perspective, parents are often considered the family members who set the tone for the family environment, the type of parenting adolescents receive is an important factor to consider in their relationships with their family members (Milevsky et al., 2011). In addition, this may be especially important in families with two or more children, as family systems theory asserts that all family sub-systems (e.g., parent-child, sibling) and individuals impact the functioning and well-being of all other sub-systems and individuals (Cox and Paley, 1997). Previous research has generally indicated that treating siblings equally is good for children (Coldwell et al., 2008), but parents also indicate that it is difficult to do so (Blake, 1981), often due to the different temperament siblings display even though they share similar environments (Dunn and Plomin, 1991). Thus, the primary aim of the present study is to examine the interactive effect of parenting similarity and sibling temperament similarity on the quality of the relationships among the family members.

### Positive Family Relationships

Experiencing positive relationships by adolescents with both parents and siblings is related to positive developmental outcomes, including fewer emotional and behavioral problems (Flouri et al., 2016; Guimond et al., 2016), more prosocial behaviors (Ferreira et al., 2016), better academic achievement (Caputi et al., 2017), and increased well-being (Fabricius et al., 2012). Scholars indicate that parent-child closeness and parental affection are associated with adolescent self-worth. Promoting a positive parent-child relationship would increase adolescent self-worth, thereby promoting adolescent mental health in turn (Mcadams et al., 2017). Longitudinal data also reveal that sibling affection is positively related to self-regulation and prosocial behaviors and negatively related to externalizing behaviors; while sibling hostility is positively related to internalizing behaviors (Buist, 2010; Padilla-Walker et al., 2010). Given that better family relationships will promote the well-being and development of adolescents, a key question is “how can relationships among family members be improved?” Thus, this study will explore how parenting similarity and temperament similarity affect positive family relationship qualities.

### Differences and Similarities in Parenting Within Families

Parenting is an important factor impacting the quality of relationships with family members. For example, positive maternal and paternal parenting styles (e.g., acceptance and behavioral control) are associated with positive representations of family relationships (Shamir et al., 2001; Milevsky et al., 2011). In the case of families with multiple children, parents often espouse loving their children equally but treating children equally can be difficult (Jeong, 2013). Parental differential treatment is linked to negative child and adolescent outcomes (Barrett Singer and Weinstein, 2000; Boyle et al., 2004; Coldwell et al., 2008). Social comparison theory (Festinger, 1954) has been used to explain the association between parental differential treatment and psychopathology of children and adolescents (Buist et al., 2013). Children compare the treatment they get with the treatment their siblings receive, which may cause feelings of unfairness, personal insecurity and anxiety (Boyle et al., 2004), lower self-esteem and sibling positivity (McHale et al., 2000), and more problematic behaviors (Richmond et al., 2005; Jeannin and Leeuwen, 2015).

Despite studies indicating negative effects of parental differential treatment, other studies indicate that differential treatment does not always lead to negative outcomes. For example, although Kowal and Kramer (1997) point out that children perceive parental differential treatment in one-third of the instances, 75% of these children do not find this to be “unfair.” If children justify differential parental behaviors by identifying ways that they and their sibling differed from one another (such as age, personal attributes, needs, relationship with parents, or strategic behaviors), they generally experience more positive appraisals about their sibling relationship. Other studies of families with a child on the autism spectrum or other disabilities reveal similar results (Stoneman, 2005; Chan and Goh, 2014). Although parents pay more attention to the child with health or developmental concerns, this differential treatment does not have negative effects on their siblings (Boll et al., 2005). The negative influence of differential parenting may be reduced, then, if adolescents and their siblings have different parenting needs.

### Differences and Similarities in Sibling Temperament

Children growing up within the same family can be remarkably different in their temperament and personalities (Dunn and Plomin, 1991; Hetherington et al., 1994), and the temperaments of children have been found to elicit different parenting. For example, low child positivity is associated with low maternal affection, and negative emotionality is associated with high psychological and behavioral controlling attempts of mothers (Laukkanen et al., 2014). In addition, these findings suggest that if siblings have different temperaments, or behave in different ways more generally, they may elicit different types of parenting.

Sibling deidentification theory suggests that siblings can purposefully try to act differently from their siblings with increasing age, which may elicit different parenting (Scarr and Grajek, 1982; Whiteman et al., 2007). Older siblings believe themselves to be more mature and responsible and do not want to be “babied” like they think their younger sibling still should be (Bumpus et al., 2001), While, younger siblings may actively try to be different from their older siblings to garner their unique parental attention or standing within the family.

Therefore, parents may have more family-wide ways of parenting or more child-specific ways of parenting, and this may be interactive with temperaments of siblings, which could be more similar within the family, or more different and child-specific. From a behavioral genetics perspective (Hetherington et al., 1994), non-twin biological siblings will share some heritable characteristics, and sharing similar living environments may also increase the likelihood that siblings may develop some similar temperamental tendencies; however, the non-shared environment (e.g., peer relationships and activity involvement) may lead to different sibling temperaments and behaviors, particularly as youth move into adolescence and seek out greater experiences outside of the family. Whether parents respond to similar and different temperamental characteristics of children in family-wide, more similar ways, or in child-specific ways will likely impact the quality of the parent-child and sibling relationships.

### The Present Study

In summary, the present study attempts to examine the interactive effect of parenting similarity and temperament similarity on positive family relationship qualities siblings experience with both their parents and one another. Based on the above discussion, we hypothesize that sibling temperament similarity will moderate the association between parenting similarity and relationship positivity with family members.

## Method

### Participants

Participants included firstborn children, who were 12–18 years old (*M* = 14.97 and *SD* = 1.68), and their next younger siblings, who were 9–17 years old (*M* = 12.20 and *SD* = 1.92 years), from 145 families with at least two children and one parent. The mean age difference between older and younger siblings was 2.77 years. The majority of families reported their ethnicity as European American (91.7%), with other families indicating African American (5.6%) or other ethnicities (2.8%). Parents who were married and the birth parents of study siblings made up 74.8% of the sample (14.7% single, divorced, or separated), while 72.3% of parents had at least a college degree. Median family income ranged between $70,000 and 84,000 (13.2%). The sample was slightly higher in percentage European American and higher in median family income than the overall community it was selected from, but relatively evenly distributed among the four possible sibling gender compositions (brother-brother *n* = 37, sister-sister *n* = 43, older brother-younger sister *n* = 28, and older sister-younger brother *n* = 37). All 145 families participated in various tasks in a laboratory setting at Time 1 (2008–2009), and data from 126 families were collected *via* online surveys 1 year later at Time 2 (2009–2010) an attrition rate of 13%), which could be completed at home. Independent samples *t*-tests were conducted to compare the families who participated at both time points with those who did not on ethnicity, marital status, education, income, as well as study variables at Time 1. The results showed that there were no significant differences between retained and attrited families, data were likely to be missing at random (MAR).

### Procedure

Participants were recruited from six junior high and high schools within a Midwestern college town in the United States of ~100,000 residents. Letters were sent to 3,030 families with an adolescent in 8th, 10th, or 12th grade. Families who were interested in the study and met the inclusion criteria described in the letter (i.e., older one must be the firstborn children in their families and have a second eldest sibling) called or e-mailed the investigators to schedule a 2 h visit in the lab. At least one parent from every family had to attend the lab visit with the two oldest children of the family and was paid honoraria for participating. As part of a larger study, family members completed questionnaires, interviews, and participated in videotaped discussion tasks together at Time 1 (2008–2009). At Time 2 (2009–2010), given that families were only providing questionnaire data and not needed for in-person interviews or discussion tasks, families completed online follow-up surveys. Only questionnaire data was used in the present study.

### Measures

All measures employed in the present study were self-reports of older and younger siblings.

#### Temperament Similarity

The Adolescent Temperament Questionnaire, a 54-item measure (Scheier et al., 1995), was used to assess a holistic view of adolescent temperament at Time 1. The measure included 11 constructs: mood, adaptability, approach/withdrawal, activity, rhythmicity, threshold, intensity, persistence, distractibility, and ego control. Older and younger siblings rated their perceptions of their temperaments on a 5-point Likert scale ranging from 1 (never) to 5 (always). Some sample items included, I can easily talk with strangers, and I fidget during quiet activities. Cronbach alphas across all subscales ranged from 0.39 to 0.81 (refer to Table 1).

**Table 1:** The Cronbach alphas of temperament for both older and younger sibling.

**Temperament**	**Older child**	**Younger child**
1. Adaption	0.746	0.735
2. Approach/withdrawal	0.647	0.505
3. Activity	0.690	0.648
4. Rhythmicity	0.657	0.716
5. Threshold	0.802	0.780
6. Intensity	0.522	0.392
7. Persistence	0.496	0.484
8. Distractibility	0.671	0.689
9. Positive mood	0.743	0.789
10. Negative mood	0.805	0.763
11. Ego control	0.658	0.679

Euclidean distance was the most popular distance measure for multidimensional scaling, then the similarity is inversely related to distance (Ashby and Ennis, 2007), thus, we calculated Euclidean distance score (*d*(p, q)= ([(p_1_-q_1_)^2^+(p_2_-q_2_)^2^+.+(p_n_-q_n_)^2^]^1/2^) of every subscale for each pair to measure the temperament similarity between the older and younger siblings in each dyad, then, we used 1/(*d*+1) to represent the similarity of temperament between older and younger siblings (Ashby and Perrin, 1988). Higher scores indicated more similarity between the temperament of siblings.

#### Parenting Similarity

The Child's Report of Parental Behavior Inventory (CRPBI) was a commonly used scale to assess general parenting behaviors at Time 1 (Schaefer, 1965; Schludermann and Schludermann, 1988). The 30-item version was completed by both older and younger siblings about their mothers and fathers separately. The measure assessed three dimensions of parenting: acceptance vs. rejection, psychological control vs. autonomy, and firm vs. lax control. Acceptance vs. rejection assessed the emotional aspects of parenting, i.e., displays of warmth and support. Psychological control vs. autonomy assessed the other aspect of psychological control that is characterized by more coercive behaviors. Firm vs. lax control assessed the behavioral control used by parents. Children rated each item on a 3-point Likert scale: the reported behavior was “like,” “somewhat like,” or “not like” about the behavior of their parent. The Cronbach's alphas of the perceived paternal and maternal parenting as reported by both children ranged from 0.53 to 85.

Similar to temperament, we also calculated Euclidean distance across siblings (*d*), and *1*/(*d*+1) was again used to compute the similarity of each parenting subscale for both fathers and mothers.

#### Positive Relationship Quality

The Network of Relationships Inventory (NRI; Furman and Buhrmester, 1985) included 39-items and was used to assess the quality of the sibling relationship and the relationship quality with mother and father at Time 1 and Time 2. The measure included 13 relationship quality subscales that load on three factors as shown in previous research (Adams and Laursen, 2007). For the present study, we were only interested in positive features of each relationship as assessed by 24 items across 8 sub-scales (assessed at Times 1 and 2): companionship, instrumental aid, intimacy, nurturance, affection, admiration, reliable alliance, and support. Items were scored on a 5-point Likert-type scale from 1 (little or none) to 5 (the most). Sample items included “How much free time do you spend with this person?” Each sibling responded to the scales about their sibling, mother, and father. Cronbach's alpha values for Times 1 and 2 were ranged from 0.62 to 0.94.

### Analytical Strategy

To examine the hypothesized moderating effects of temperament similarity in the link between parenting similarity and positive family relationship qualities, we conducted a series of latent moderator structure models (LMS) with a maximum likelihood estimation method using the Mplus statistical software that estimated parameters incorporating full information maximum likelihood (FIML) methods. The FIML methods allow data from all individuals to be included regardless of their pattern of missing data and are more appropriate than other commonly used methods (Arbuckle, 1996). Using LMS models offered an advantage by partitioning the true scores from the residual variances in observation variables and producing optimally reliable latent scores. All models in the present study were standardized.

The latent moderated structural equations approach does not yield the standard Chi-square and related model fit statistics. Instead, the Mplus program yields information criteria indices, such as the sample-size adjusted Bayesian information criterion (SSABIC) and the Akaike information criterion (AIC). Lower values reflect better model fit (Klein and Moosbrugger, 2000).

## Results

### Bivariate Analyses

Table 2 shows means, SD, intercorrelations for studied variables, as well as sibling gender composition (coded same-gender as 1 and mixed-gender as 2), and age of the adolescents.

**Table 2:** Descriptive statistics and inter-correlations of the variables.

		**Time 2 positive relationship quality**							
		**Older perspective**			**Younger perspective**			***M***	***SD***
		**Sibling**	**Child-Mother**	**Child-Father**	**Sibling**	**Child-Mother**	**Child-Father**		
Gender composition		−0.21^*^	0.14	−0.03	−0.15^+^	0.01	0.03	1.45	0.50
Older siblings' age		−0.02	0.05	0.05	−0.20^*^	−0.37^***^	−0.11	14.97	1.68
Younger siblings' age		−0.04	−0.03	0.10	−0.24^**^	−0.41^***^	−0.13	12.20	1.92
	P1	0.20^*^	0.15	0.14	0.10	0.18^*^	0.11	0.31	0.10
Maternal parenting	P2	0.06	0.03	0.11	0.04	−0.07	0.01	0.29	0.09
	P3	0.14	0.12	0.09	0.12	0.13	0.01	0.31	0.09
	P1	0.09	−0.02	0.16^+^	0.00	0.01	0.14	0.30	0.10
Paternal Parenting	P2	−0.06	−0.15	−0.10	0.07	−0.17^+^	−0.14	0.30	0.10
	P3	0.07	−0.02	0.07	−0.09	−0.19^*^	−0.01	0.30	0.07
	Temp 1	0.03	−0.06	0.16^+^	−0.11	−0.11	0.08	0.33	0.12
	Temp 2	0.23^*^	0.02	0.13	0.03	−0.08	−0.03	0.28	0.08
	Temp 3	0.04	0.03	0.08	0.11	0.03	0.09	0.27	0.10
	Temp 4	−0.03	0.02	0.07	−0.15	−0.19^*^	−0.02	0.45	0.22
	Temp 5	−0.05	−0.06	−0.09	−0.09	−0.13	−0.06	0.25	0.07
Temperament	Temp 6	0.03	0.02	−0.05	0.01	0.05	0.05	0.26	0.10
	Temp 7	0.07	0.08	0.07	0.06	−0.01	0.04	0.28	0.08
	Temp 8	−0.01	−0.16^+^	−0.12	0.01	−0.01	−0.10	0.24	0.07
	Temp 9	0.05	0.09	0.04	−0.03	0.00	0.08	0.31	0.14
	Temp10	−0.13	−0.09	−0.01	−0.01	0.00	0.06	0.25	0.07
	Temp11	0.03	−0.01	0.01	0.03	−0.12	−0.02	0.27	0.10
*M*		3.01	3.40	3.16	3.09	3.59	3.30	–	–
*SD*		0.79	0.76	0.78	0.89	0.77	0.81	–	–

*P1–P3 three dimensions of parenting; Temp1–Temp11 11 dimensions of temperament*.

+ *p < 0.10*,

* *p < 0.05*,

** *p < 0.01*,

*** *p < 0.001*.

The results indicated that sibling gender composition was related to a lower level of positive sibling relationship quality (*r*_older_ = −0.21, *p*_older_ < 0.05; *r*_younger_ = −0.15, *p*_younger_ < 0.10). For younger siblings, greater age was related to a lower level of positive sibling relationship quality (*r*_older_ = −0.20, *p* < 0.05; *r*_younger_ = −0.24, *p* < 0.01) and child-mother relationship quality (*r*_older_ = −0.37, *p* < 0.001; *r*_younger_ = −0.41, *p* < 0.001). A higher level of maternal parenting similarity was related to higher positive sibling relationship quality (“acceptance vs. rejection,” *r*_older_ = 0.20, *p*_older_ < 0.05; *r*_younger_ = 0.18, *p*_younger_ < 0.05). Similarly, a higher level of paternal parenting similarity was related to higher positive older-father relationship quality (“acceptance vs. rejection,” *r* = 0.16, *p* < 0.10) and lower level of positive younger-mother relationship quality (“psychological control vs. autonomy,” *r* = −0.17, *p* < 0.10; and ‘firm vs. lax control'; *r* = −0.19, *p* < 0.05). Temperament similarity was also proved to be correlated to positive relationship qualities significantly, higher level of “adaptive” similarity was related to higher level of positive older-father relationship quality (*r* = 0.16, *p* < 0.10), “approach vs. withdrawal” similarity was related to higher level of positive older-younger sibling relationship quality (*r* = 0.23, *p* < 0.05), “rhythmicity” similarity was related to lower level of positive older-mother relationship quality (*r* = −0.16, *p* < 0.10); higher level of “threshold” similarity was related to lower level of positive younger-mother relationship quality (*r* = −0.19, *p* < 0.05).

### Moderation Models

**Figure 1:**
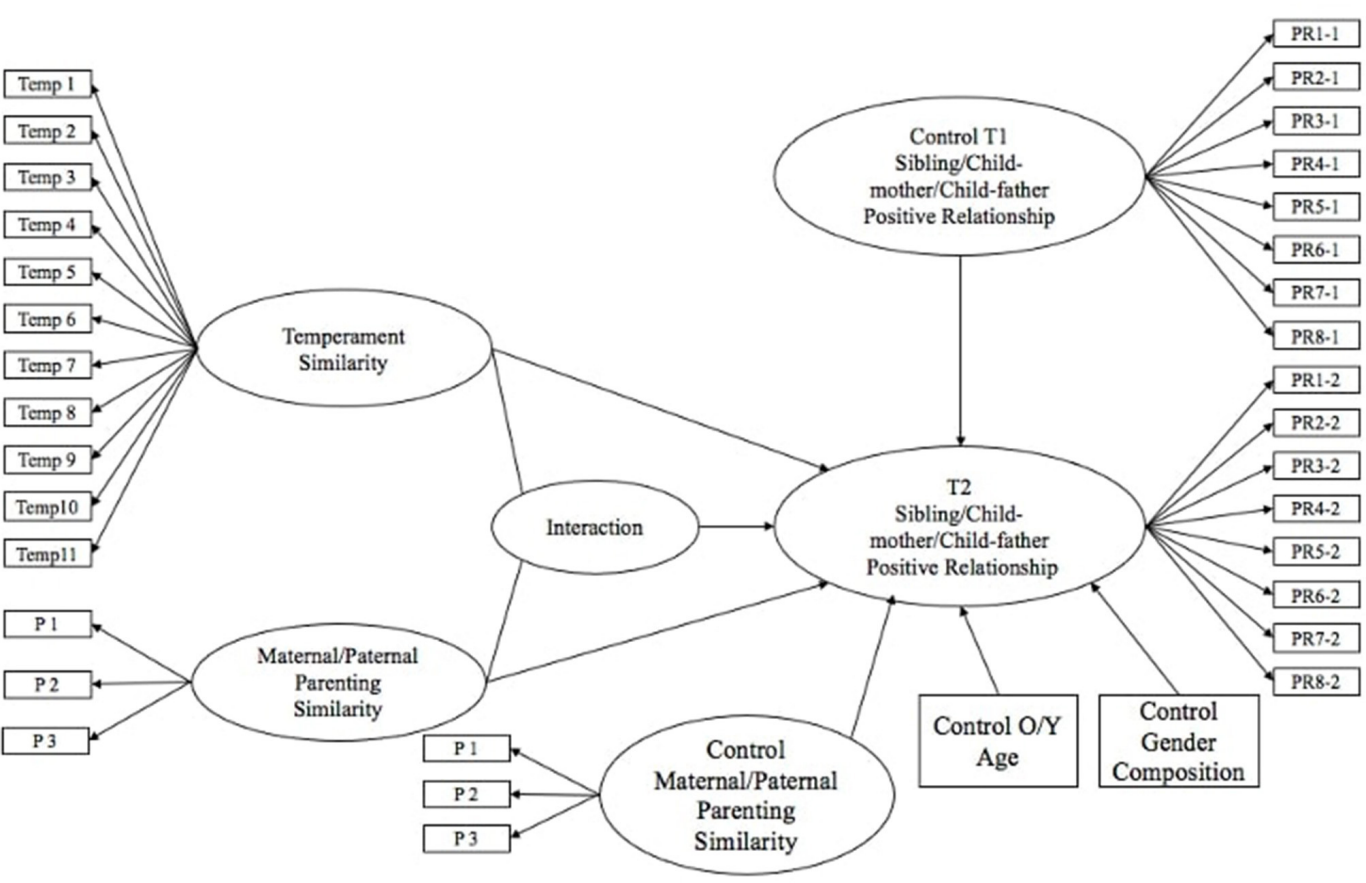
Hypothesized Latent Moderator Structure Model. PR1-PR8 eight dimensions of positive relationship quality.

To examine the effect of temperament similarity and paternal/maternal parenting similarity on the Time 2 positive relationship qualities, we conducted a series of moderation analyses with the separate LMS for positive relationship qualities of older and younger siblings with their siblings and parents (refer to Figure 1). Thus, there were eight models: maternal parenting similarity and temperament similarity predicting positive sibling relationship quality as perceived by older and by younger siblings (Models 1–2), paternal parenting similarity and temperament similarity predicting positive sibling relationship quality as perceived by older and younger sibling (Models 3–4), maternal parenting similarity and temperament similarity predicting positive child-mother relationship quality as perceived by older and younger sibling (Models 5–6), and paternal parenting similarity and temperament similarity predicting positive child-father relationship quality as perceived by older and younger sibling (Models 7–8). Finally, each model was controlled for adolescent age, gender composition, Time 1 positive relationship quality, and the parenting similarity of other parents. In each model, we examined the main effect of parenting similarity and temperament similarity and the interaction effects between parenting similarity and temperament similarity. The significant effects are shown in Table 3 and are described below.

**Table 3:** Predicting positive family relationship at Time 2.

	**Unstandardized parameter estimate (** ***SE*** **)**							
	**Positive**				**Positive**		**Positive**	
	**sibling relationship**				**child-mother relationship**		**child-father relationship**	
**Parameter**	**Model 1**	**Model 2**	**Model 3**	**Model 4**	**Model 5**	**Model 6**	**Model 7**	**Model 8**
Gender composition	−0.060	−0.081	−0.055	−0.079	0.086	−0.037	−0.032	−0.012
Age	0.076	−0.235^**^	0.078	−0.233^**^	0.121^+^	−0.371^***^	0.074	−0.094
Positive relationship (Time 1)	0.878^***^	0.562^***^	0.864^***^	0.553^*^	0.909^***^	0.274	0.669^***^	0.696^***^
Maternal parenting similarity	−0.043	0.022	0.050	0.046	−0.204^+^	0.728	0.318^***^	0.297
Paternal parenting similarity	0.220^***^	0.001	0.122	−0.018	0.161^**^	−0.856	−0.113	−0.505^***^
Temperament similarity	−0.111	−0.104	−0.084	−0.102	−0.054	0.107	−0.195	0.056
Maternal parenting similarity × Temperament similarity	0.117^*^	0.053^*^			0.058^*^	0.002		
Paternal parenting similarity × Temperament similarity			0.109^*^	0.056^+^			0.174^*^	0.048^+^

+ *p < 0.10*,

* *p < 0.05*,

** *p < 0.01*,

*** *p < 0.001*.

### Maternal Parenting Similarity and Sibling Temperament Similarity as Predictors of Positive Sibling Relationship Quality

First, we examined the effects of maternal parenting similarity and temperament similarity of siblings on Time 2 reports of adolescents of positive sibling relationship quality while control for paternal parenting similarity, Time 1 positive sibling relationship quality, gender composition, and adolescent age. Separate models were computed for the older sibling and the younger sibling.

The results are presented first for the model for the older sibling (Model 1, AIC = 327.384, BIC = 660.778, and SSABIC = 306.370). The interaction between temperament similarity and maternal parenting similarity in siblings was significant (β = 0.117 and *p* = 0.027). Simple slopes were used to interpret the significant interaction effect, when siblings were high on temperament similarity, a higher level of maternal parenting similarity reported by older siblings was related to a higher level of positive relationship quality with their younger siblings (*B* = 40.217, *SE* = 1.389, *t* = 28.951, and *p* < 0.001). In contrast, when siblings were low on temperament similarity, a higher level of maternal parenting similarity reported by older siblings was also related to a lower level of positive relationship quality with their younger siblings (*B* = −41.473, *SE* = 1.570, *t* = −26.422, and *p* < 0.001).

Next, we examined the model for younger siblings (Model 2, AIC = 686.486, BIC = 1019.880, and SSABIC = 665.472). The interaction (maternal parenting similarity × temperament similarity) effect on Time 2 positive sibling relationship quality was also significant (β = 0.053 and *p* = 0.012). To interpret the significant interaction effect, simple slopes were estimated, when siblings were high on temperament similarity, a higher level of maternal parenting similarity reported by younger siblings was related to a higher level of positive relationship quality with siblings (*B* = 23.433, *SE* = 9.665, *t* = 2.424, and *p* = 0.015). In addition, when siblings were low on temperament similarity, a higher level of maternal parenting similarity reported by younger siblings was also related to a lower level of positive sibling relationship quality (*B* = −22.599, *SE* = 9.554, *t* = −2.365, and *p* = 0.018).

### Paternal Parenting Similarity and Sibling Temperament Similarity as Predictors of Positive Sibling Relationship Quality

Similar to above, we examined the effects of paternal parenting similarity and temperament similarity of siblings on Time 2 sibling reports of adolescents positive sibling relationship quality while control for maternal parenting similarity, Time 1 positive sibling relationship quality, gender composition, and older/younger age. Separate models were computed for older and younger siblings.

The interaction effect (paternal parenting similarity × temperament similarity) on Time 2 positive sibling relationship quality was significant (β = 0.109 and *p* = 0.031) for older siblings (Model 3, AIC = 327.836, BIC = 661.230, and SSABIC = 306.822) and marginally significant for younger siblings (β = 0.056, *p* = 0.052; Model 4, AIC = 686.440, BIC = 1019.834, and SSABIC = 665.426). Simple slopes analyses for model 3 showed that when siblings were high on temperament similarity, paternal parenting similarity was related to positive sibling relationship quality at Time 2 positively (*B* = 48.128, *SE* = 1.896, *t* = 25.382, and *p* < 0.001), while paternal parenting similarity was related to positive sibling relationship quality at Time 2 negatively (*B* = −43.763, *SE* = 2.514, *t* = −17.405, and *p* < 0.001). However, the simple slopes analyses for model 4 showed that whether siblings were high or low on temperament similarity, paternal parenting similarity was not significantly related to positive sibling relationship quality at Time 2 (*B*_*high*_ = 30.394, *SE*_*high*_ = 19.783, *t*_*high*_ = 1.536, *p*_*high*_ =0.124; *B*_*low*_ = −31.259, *SE*_*low*_ = 21.418, *t*_*low*_ = −1.459, *p*_*low*_ = 0.144).

### Maternal Parenting Similarity and Sibling Temperament Similarity as Predictors of Positive Child-Mother Relationship Quality

We also conducted two more models to examine how temperament similarity of siblings and maternal parenting similarity affected positive child-mother relationship quality and control for paternal parenting similarity, Time 1 positive child-mother relationship quality, gender composition, and older/younger age. Only the model for older siblings (Model 5, AIC = 349.556, BIC = 682.950, and SSABIC = 328.542) showed a significant main effect of maternal parenting similarity (β = −0.204 and *p* = 0.087) and a significant interaction (β = 0.058 and *p* = 0.019). Simple slopes analyses found that when siblings were high on temperament similarity, maternal parenting similarity was related to positive child-mother relationship quality at Time 2 positively (*B* = 12.380, *SE* = 5.524, *t* = 2.241, and *p* = 0.025), while maternal parenting similarity was related to positive child-mother relationship quality at Time 2 negatively (*B* = −16.878, *SE* = 5.080, *t* = −3.322, and *p* = 0.001).

### Paternal Parenting Similarity and Sibling Temperament Similarity as Predictors of Positive Child-Father Relationship Quality

Finally, we examined the effects of paternal parenting similarity and temperament similarity of siblings on Time 2 reports of adolescents of positive child-father relationship quality while control for maternal parenting similarity, Time 1 positive child-father relationship quality, gender composition, and older/younger age. Both the models for older and younger siblings had significant main effects and interaction effects. The result of the model for older siblings (Model 7, AIC = 360.343, BIC = 693.737, and SSABIC = 339.329) revealed a significant interaction effect on child-father relationship positivity at Time 2 (β = 0.174 and *p* = 0.029). Simple slope analyses indicated that when siblings were high on temperament similarity, a higher level of paternal parenting similarity reported by older siblings was related to a higher level of positive relationship qualities with their fathers (*B* = 68.258, *SE* = 19.075, *t* = 3.578, and *p* < 0.001); when siblings were low on temperament similarity, a higher level of paternal parenting similarity reported by older siblings was related to lower level of positive relationship qualities with their fathers (*B* = −71.987, *SE* = 18.471, *t* = −3.897, and *p* < 0.001).

We also discovered a significant main effect of paternal parenting (β = −0.505 and *p* < 0.001) and a marginally significant interaction effect (β = 0.048 and *p* = 0.097) in the model for younger siblings (Model 8, AIC = 179.391, BIC = 512.785, and SSABIC = 158.377). Simple slope analyses indicated that when siblings were high on temperament similarity, a higher level of paternal parenting similarity reported by younger siblings was related to a higher level of positive relationship qualities with their fathers (*B* = 13.943, *SE* = 9.630, *t* = 1.448, and *p* > 0.05); when siblings were low on temperament similarity, a higher level of paternal parenting similarity reported by older siblings was related to a lower level of positive relationship qualities with their fathers (*B* = −35.730, *SE* = 10.907, *t* = −3.276, and *p* = 0.001).

## Discussion

Previous research on parental differential treatment of siblings has typically found it to be detrimental to youth adjustment and their relationships (Brody et al., 1992, 1998); however, psychologists also encourage parents to be sensitive in their responses to needs and temperamental characteristics of individual children (Sanson and Rothbart, 1995; Bates et al., 2012). While these issues are relatively easy to consider individually when parents are attempting to make “real life” parenting decisions in families with multiple children, it can be difficult to decide the best way to proceed. Therefore, in the present study, we examined the effect of parenting similarity and temperament similarity on positive relationship qualities of siblings with family members from the framework of family systems theory (Cox and Paley, 1997). In general, these findings revealed that temperament similarity had a moderating effect on the association between parenting similarity and perceived relationship positivity of adolescents with all family members, with only two exceptions: sibling temperament similarity did not moderate the effects of paternal parenting similarity on positive sibling relationship quality and maternal parenting similarity on positive child-mother relationship quality for younger siblings. In general, when children were high in temperament similarity, higher levels of parenting similarity with mothers or fathers were related to more positive relationship qualities with their siblings, mothers, and fathers 1 year later; however, lower levels of maternal or paternal parenting similarity were related to less positive relationship qualities with siblings, mothers, and fathers 1 year later. Furthermore, when children were low on temperament similarity, higher levels of parenting similarity with mothers or fathers were generally related to less positive relationship qualities with family members, while lower levels of parenting similarity were related to more positive relationship qualities with family members.

### Temperament Similarity and Similar Parenting

Within the same biological family, siblings share similar genes, environments, and resources; however, comparison of parenting may cause feelings of unfairness, which may, in turn, exert influence on family relationships and dynamics (Coldwell et al., 2008). Consistent with this notion, the results of the present study showed that similar parenting, not different parenting, was the most positive for family relationship functioning when siblings had similar temperaments to one another.

From the perspective of psychological needs and social comparison theory (Festinger, 1954), if children are more temperamentally similar, they may require having similar needs met by parents. Parental differential treatment, then, may cause unfair feelings, and lead to reduced relationship positivity. Consistent with previous studies about the links between differential treatment of parents and sibling relationships, less parental differential treatment is associated with better self-esteem of children (McHale et al., 2000), adjustment (Dunn et al., 1990; McHale and Pawletko, 1992; Feinberg and Hetherington, 2001), and decreased negative emotionality (Brody et al., 1992). On the contrary, adolescents, who experience less parental warmth or more conflict relative to their siblings, exhibit less sibling warmth and more conflict, even when controlling for their dyadic relationships with mothers and fathers (Shanahan et al., 2008). Although adolescents may not recognize these underlying feelings, overt negativity toward a sibling may be grounded in rivalry and jealousy that emerge when youth believe their siblings are favored by parents (McHale et al., 2016).

### Temperamental Differences and Different Parenting

We also found that different parenting, not similar parenting, was better for family relationship quality in the context of differently tempered siblings. Behavioral geneticists point out that two siblings growing up together are often no more genetically alike than unrelated youth (Rowe, 1990; Dunn and Plomin, 1991). Along with shared history and intimate knowledge of a sibling, individuals are also motivated to carve out unique identities, which may be shaped, in part, by their perception of their attributes and qualities of siblings (Whiteman et al., 2007). Thus, Schachter et al. (1976) proposed the concept of “sibling deidentification,” a tendency for siblings to consciously or unconsciously define themselves as different from one another to reduce competition, which has been regarded as a way to protect siblings from social comparison and rivalry.

Theory and previous research suggest that children may have a greater need to differentiate from siblings who are more similar to them in contexts, such as gender (Brody et al., 1998). Therefore, parents need to be sensitive to own needs of each of their children, exert appropriate parenting for him/her (Kowal and Kramer, 1997), which in turn will promote relationship positivity within families, which is consistent with these results. In addition, studies show that parents have an easier time recognizing and admitting to differential treatment when their two children are different (e.g., gender and age), but recognition of different temperaments is often neglected (Crouter et al., 1999). Therefore, the present study filled this gap.

### The Effect on Different Family Relationships

The pattern of findings also revealed some differences across relationships with mothers, fathers, and siblings than hypothesized. More specifically, two findings did not align with past research and social comparison theory. The main effect of maternal parenting similarity was negatively associated with relationship positivity of older siblings with mothers, while the main effect of paternal parenting similarity was negatively associated with relationship positivity of younger siblings with fathers. Older siblings in this adolescent sample are particularly invested in their growing autonomy, which likely makes them prefer not to be treated similarly to their younger siblings because they believe themselves to be more mature and responsible (Small et al., 1988; Bumpus et al., 2001). On the contrary, younger siblings may hope to show their uniqueness in comparison to their older siblings (Grunwald and McAbee, 1999), and this may be particularly the case with fathers as fathers become more involved in caretaking with the addition of more than one child to the household (Yeung et al., 2001).

Additionally, the hypothesized interactions between maternal parenting similarity, temperament similarity, and perceptions of the mother-child relationship by younger siblings, as well as between paternal parenting similarity, temperament similarity, and perceptions of the sibling relationship by younger siblings were not supported due to non-significant associations. Given that the majority of the tested interactions did reveal significant results, and in general in the hypothesized direction, it is difficult to know whether these lack of associations are meaningful or spurious.

### Limitations, Future Directions, and Conclusion

Despite the strengths of the multi-informant, multi-relationship, longitudinal design, this study has several limitations. First, the sample we used in this study was a predominantly White, middle-class, and North American sample. Future studies need to examine if the same effects will be present in other cultural contexts. For example, while traditionally many Chinese adolescents grow up as only children, with the relatively recent opening of a two-child policy, a key question for parents in this cultural context is how to balance the needs of multiple children while still providing appropriate parental attention for each child. Second, sibling gender composition may be particularly influential in these processes. For example, previous research indicates that mothers and fathers report having more conflictual relationships with their children from same-gender dyads than with children from opposite-gender dyads (Stocker, 1995; Jenkins et al., 2003). In the present study, we were able to control for sibling gender composition, but future research would require a larger sample to examine multi-group comparisons across the four possible gender compositions (i.e., sister-sister, brother-brother, brother-sister, and sister-brother). Third, given only a one-time point of measurement for temperament and parenting similarity, it is hard to tell whether temperament similarity conditions the effects of parenting similarity or it is the outcome of parenting similarity. Future studies would benefit from collecting data from multiple time points to confirm the direction of the effects. Last, due to the fairly small sample, including more variables in the model may encounter model under-identification or non-convergence issues, so the present study did not control some important demographic covariates, such as the age gap of siblings and socioeconomic resources of families. Future studies need to enlarge the sample size to study the relationship among parenting similarity, temperament similarity, and positive family relationship.

Most families in the US have more than one child, so the investigation and exploration of parenting, temperament, and family relationship quality are imperative to guide parents on how to raise their children. In general, these findings suggest that parents of multiple children should focus more on being sensitive to the individual needs of their children, rather than be concerned about treating their children equally at all costs. Such information may be beneficial to not only parents but also clinicians and interventionists who work with families.

## Data Availability Statement

The data analyzed in this study is subject to the following licenses/restrictions: Data was only originally approved to be allowed for use by members of Campione-Barr lab. Requests to access these datasets should be directed to campionebarrn@missouri.edu.

## Ethics Statement

The studies involving human participants were reviewed and approved by University of Missouri-Columbia, Institutional Review Board. Written informed consent to participate in this study was provided by the participants' legal guardian/next of kin.

## Author's Note

A previous version of this study was presented at the Nov. 2019 National Council on Family Relations meetings in Fort Worth, TX.

## Author Contributions

XS contributed by conceptualizing study, analyzing data, and writing manuscript. NC-B contributed by funding data collection, recruiting families, collecting data, cleaning data, conceptualizing study, and writing some portions of the manuscript. All authors contributed to the article and approved the submitted version.

## Conflict of Interest

The authors declare that the research was conducted in the absence of any commercial or financial relationships that could be construed as a potential conflict of interest.

## Publisher's Note

All claims expressed in this article are solely those of the authors and do not necessarily represent those of their affiliated organizations, or those of the publisher, the editors and the reviewers. Any product that may be evaluated in this article, or claim that may be made by its manufacturer, is not guaranteed or endorsed by the publisher.
